# The Change in Distance Between Bilateral Internal Carotid Arteries in Acromegaly and Its Risk Factors

**DOI:** 10.3389/fendo.2020.00429

**Published:** 2020-07-07

**Authors:** Xiaorong Yan, Xiaoyong Chen, Hongliang Ge, Shinong Zhu, Yuanxiang Lin, Dezhi Kang, Zhangya Lin, Changzhen Jiang, Chenyu Ding

**Affiliations:** ^1^Department of Neurosurgery, The First Affiliated Hospital of Fujian Medical University, Fuzhou, China; ^2^Department of Neurosurgery, Jinjiang Hospital Jinnan Branch Courts, Jinjiang, China

**Keywords:** refractory pituitary adenoma, internal carotid artery, acromegaly, disease duration, computed tomography angiography, magnetic resonance angiography

## Abstract

**Background:** Studies investigating the change in distance between the bilateral internal carotid arteries (ICAs) in acromegalic patients have provided ambiguous results. The influencing factors of these changes have not been well-identified.

**Objective:** To further investigate the change in distance between bilateral ICAs in acromegaly patients and identify the influencing factors of the change.

**Method:** Patients diagnosed as acromegaly from Jan 2016 to Sep 2019 in the Department of Neurosurgery of the First Affiliated Hospital of Fujian Medical University, were included in this study. Computed tomography angiography (CTA) or magnetic resonance angiography (MRA) data were obtained for all patients for three-dimensional reconstruction of the ICAs. Distance between bilateral ICAs was measured and recorded for assessment.

**Result:** 172 patients including 86 cases with acromegaly in the study group and 86 cases with non-functional pituitary adenoma in the control group were enrolled in this study. The difference of adenoma sizes between two groups was not statistically significant. Patients in acromegaly group had significantly larger maximum distances between bilateral siphon carotid ectasias (25.5 ± 4.1 vs. 23.4 ± 3.5 mm, *P* = 0.001) and between bilateral lacerum segments (26.2 ± 3.2 vs. 24.1 ± 4.3 mm, *P* < 0.001) compared with those of patients with non-functional pituitary adenomas. Multivariate analysis showed that the increased bilateral ICAs distance was associated with disease duration (odds ratio = 1.01, 95% confidence interval = 1.01–1.02, *P* = 0.005) and refractory pituitary adenoma (odds ratio = 9.8, 95% confidence interval = 1.1–88.7, *P* = 0.043) but not with level of growth hormone (GH), insulin-like growth factor-1 (IGF-1) and adenoma size in acromegaly.

**Conclusion:** Our study showed significant change in distance between the bilateral ICAs in acromegalic patients, comparing to patients with non-functional pituitary adenomas. The increased intercarotid artery distance is associated with disease duration but not with preoperative level of GH and IGF-1. Refractory pituitary adenoma and longer disease duration are the both risk factors of the increased ICAs distance in patient with acromegly.

## Introduction

Acromegaly is mainly caused by the growth hormone (GH)-secreting pituitary adenoma, and characterized by excessive GH secretion. The excessive GH stimulates the growth of various tissues and impairs the structures of the heart and great vessels, which further affects their functions. Acromegalic patients also showed a higher risk of co-morbidities such as hypertension, diabetes and hypopituitarism than general population. Furthermore, there is a higher chance for these patients to have intracranial aneurysms (1, 2), which is known as a main cause of cerebrovascular accidents (3, 4). In acromegalic patients, excessive GH has been proven a primary factor contributing to intracranial aneurysms (1), while co-morbidities, previous surgery and other clinical findings seems not related.

Several studies showed that tortuosity and ectasia of intracranial vessels were increased in acromegalic patients (5), which was associated with increased vascular mortality. Therefore, the distance between bilateral internal carotid arteries (ICAs) also changed. As we know, transsphenoidal surgery is a first-line treatment for GH-secreting pituitary adenoma, therefore, these changes were regarded as the possible risk factors for intraoperative vascular injury. Knowing detailed information of the bilateral ICAs preoperatively could impact intraoperative tumor exposure and reduce intraoperative adverse events.

However, studies investigating the changes of distance between ICAs in acromegalic patients have provided ambiguous results (5–7). Furthermore, the influencing factors of these changes have not been well-identified. With the aid of computed tomography angiography (CTA) and magnetic resonance angiography (MRA), intracranial vascular and bony structure could be modeled. Our study aimed at determining the changes of the distance between the bilateral ICAs in acromegalic patients and investigating the possible risk factors for those changes.

## Materials and Methods

### Subjects

Patients diagnosed as acromegaly from Jan 2016 to Sep 2019 in the Department of Neurosurgery of the First Affiliated Hospital of Fujian Medical University were included in this study as research subjects. Eighty six patients with acromegaly were included in this study. And 86 patients with non-functional pituitary adenoma, matched for age, gender and adenoma size were recruited in the control group.

The inclusion criteria of acromegaly group were as follows: [1] clinical diagnosis of acromegaly; [2] image diagnosis of pituitary adenoma according to contrast-enhanced magnetic resonance imaging, as well as fasting GH level higher than that of normal; [3] pathologically diagnosed as GH-secreting pituitary adenoma. Non-functional pituitary adenoma patients diagnosed by preoperative image, laboratory examination and postoperative pathology were recruited as the control group. The exclusion criteria were as follows: [1] age <18 years; [2] history of surgery, radiotherapy of other brain tumor; [3] medication of somatostatin analogs before admission to our hospital; [4] clinical, laboratory, imaging, and pathologic data set was incomplete.

The following criteria were used to diagnose acromegaly: [1] typical symptoms of acromegaly such as enlarged hand/foot and thickened soft tissue; [2] not able to lower GH to <1.0 ng/mL after oral administration of 75 g glucose; [3] a high level of fasting GH (>2.5 ng/mL); and [4] a high level of serum insulin-like growth factor-1 (IGF-1) controlled for age and gender. After documentation of their disease information, all patients were admitted, had hormone tests and CTA evaluation. Our study analyzed data of those patients. For comparison purpose, patients with non-functional adenoma, admitted during the same period in our institution were 1:1 matched for age, gender and adenoma size, and recruited as the control group. The study protocol was approved by the Ethics Committee of the hospital. The consent of all subjects was obtained in accordance with the Declaration of Helsinki.

### Data Collection and Imaging Examination

Clinical information, such as medical history (prior and current illness), admission status, image files, treatment received and other relevant hospitalization information was collected for each patient. Invasiveness of pituitary adenoma was evaluated using the Knosp classification on the enhanced MRI. Duration of disease of acromegaly was counted from the onset of typical symptoms such as enlarged hand/foot and thickening of the soft tissue. The time for headache, decreased visual acuity or identification of tumor by physical examination was recorded as duration of non-functional pituitary adenoma by medical history. The defintination of atypical adnoma and refractory adenoma was according to the 2004 WHO classification (8) and the published classification for pituitary tumors (9) as follow: Atypical adnoma: [1] elevated mitotic index (>2 mitoses per high-power field [HPF]); [2] positive p53 staining; [3] an ki-67 index >3%. Refractory adenoma: [1] tumor infltrates adjacent structures according to radiological results or intraoperative fndings; [2] tumor Ki-67 index is >3% and grow fast; [3] current treatments fail to control tumor growth and/or hormonal hypersecretion; [4] tumor recurrence occurs within 6 months after surgery.

**Figure 1:**
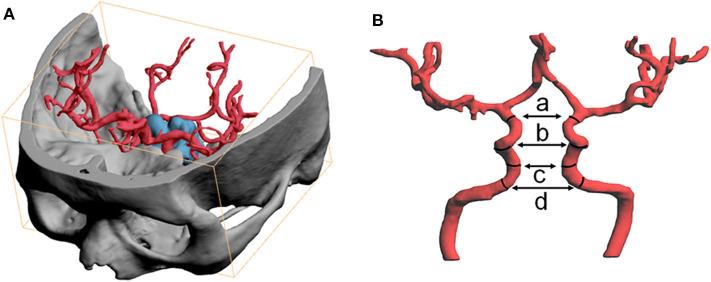
Three-dimensional image software was used to reconstruct the ICAs and to measure intercarotid distance. **(A)** The image data were exported as DICOM format file and further imported to three-dimensional image process software to reconstruct skull, pituitary adenoma and the ICAs. Fused image of skull and ICA was used to determine segments of ICA. **(B)** Measurement of intercarotid distance, parameters including (a) the distance between the inner walls of the bilateral carotids at the level of distal dural ring (ophthalmic segment); (b) the istance between ICAs at the level of the most concave point of the C4-C5 bend (siphon carotid ectasias segment); (c) the distance between ICAs at the level of posterior ascending portion of C4 segment (cavernous segment); (d) the maximum distance between ICAs at the level of C3 segment (lacerum segment).

All included patients received CTA (AquilionTM ONE systems, Toshiba, Japan) or MRA (Magneto Verio MRI system with Tim technology or Skyra MRI system, Siemens, Germany). The image data were exported as DICOM format file and further imported to three-dimensional printing software (PolyJet Studio™, Aojie, China). After reconstructing the ICAs, distance between the bilateral ICAs was measured. Based on the Bouthillier classification, parameters collected were as follows: (a). the distance between the inner walls of the bilateral carotids at the level of distal dural ring (ophthalmic segment); (b). the distance between ICAs at the level of the most concave point of the C4-C5 bend (siphon carotid ectasias segment); (c). the distance between ICAs at the level of posterior ascending portion of the C4 segment (cavernous segment); (d). the maximum distance between ICAs at the level of the C3 segment (lacerum segment). Illustration was showed in Figure 1.

### Laboratory Examination

All patients had a nine-hour fasting period before surgery for collection of serum samples. Both pituitary hormones and IGF-1 were measured according to a chemiluminescence assay (ADVIA Centaur XP, Siemens, Germany).

### Statistical Analysis

The results were expressed as mean ± standard deviation or [M(QL,QU)]. Comparisons of categorical variables were analyzed using the Chi-squared test. The 2-sample *t*-test or Wilcoxon rank-sum test was used to compare numerical variables.

The bilateral ICAs distances (a, b, c, and d, Figure 1) were compared between the acromegaly group and control group. After comparing the distances, the acromegaly group was divided into two subgroups by indicators with significant difference (distance between bilateral siphon carotid ectasias segments and distance between bilateral lacerum segments). After adding the two numbers together, patients were equally distributed into two subgroups with larger number and smaller one.

Clinical characteristics were compared to find factors related to change of ICAs distance in acromegalic patients, based on the difference of two subgroups. Multivariate logistic regression model analysis was used to assess the change of ICAs distance in patient with acromegaly. To find variables to be used in the multivariate evaluation, univariate assessment was first performed. Briefly, candidate variables for univariate logistic regression analysis included all available variables that had univariate associations *P* < 0.15 between the two acromegaly subgroups as divided by the sum of ICAs distances. All variables having *P* < 0.05 from univariate logistic regression analyses were included in multivariate analysis. Then, the final model was created using the backward stepwise multivariate regression, in which the least non-significant variables were removed from the model one at a time, until all remaining variables had *P* < 0.05. Statistical analysis was performed with SPSS version 17.0 (SPSS Inc., Chicago, Illinois). *P* < 0.05 was considered as statistically significant.

## Results

### Demographic and Clinical Data

After selection with the inclusion and the exclusion criteria, 86 cases of acromegalic patients, 46 males and 40 females, were included as study group. The mean age of patients in the acromegaly group was 40.1 ± 10.6 years. Eighty six patients with non-functional pituitary adenoma, 1:1 matched for age, gender and adenoma size, were recruited as the control group. In this series of patients, the incidences of atypical GH adenomas and refractory GH adenomas were 16.3% (14/86) and 10.5% (9/86), respectively.

The demographic information, adenoma size, laboratory results, medical history and ICAs distances of the patients are shown in Table 1. Between the two groups, there were no significant differences in gender, age, body mass index (BMI), adenoma size, smoking habit, hypertension, diabetes, and dyslipidemia.

**Table 1:** Comparison of demographic and clinical data between patients with acromegaly and non-functional pituitary adenomas.

**Characteristics**	**Acromegaly group (*n* = 86)**	**Control group (*n* = 86)**	***P*-value**
**DEMOGRAPHICS**			
Age, year	40.1 ± 10.6	41.1 ± 10.3	0.531
Gender, female	40 (46.5%)	40 (46.5%)	1.000
Height, cm	167.3 ± 6.3	165.6 ± 6.6	0.114
BMI, kg/m^2^	24.8 ± 4.1	24.2 ± 3.5	0.309
**MRI FOUNDING**			
Pituitary adenoma volume, cm^3^	2.6 (0.7, 5.4)	3.1 (0.6, 6.4)	0.418
Pituitary adenoma diameter, cm	1.7 ± 0.8	1.9 ± 1.0	0.455
Knosp grade	2 (1–3)	2 (1–3)	0.512
**LABORATORY**			
GH, μg/L	40.6 (11.4, 60.2)	0.9 (0.5, 1.4)	<0.001
IGF-1, μg/L	526.2 ± 204.4	-	-
**MEDICAL HISTORY**			
Hypertension	23 (26.7%)	18 (20.9%)	0.374
Diabetes mellitus	19 (22.1%)	16 (18.6%)	0.570
Hyperlipidemia	14 (16.3%)	13 (15.1%)	0.834
Somking history	18 (20.9%)	21 (24.4%)	0.585
Duration of disease, months	90 (48, 132)	12 (5–20)	<0.001
**BILATERAL ICA DISTANCES**			
Ophthalmic segments	16.0 ± 5.2	16.2 ± 3.4	0.730
Siphon carotid ectasias segments	25.5 ± 4.1	23.4 ± 3.5	0.001
Cavernous segments	21.9 ± 3.2	22.5 ± 2.7	0.205
Lacerum segments	26.2 ± 3.2	24.1 ± 4.3	<0.001

*Acromegaly group: patients with GH-secreting pituitary adenoma. Control group: patients diagnosed with non-functional pituitary adenomas. Values are n (%), mean (SD), median (25%−75%)*.

### Comparison of Distance Between the Bilateral ICAs

**Figure 2:**
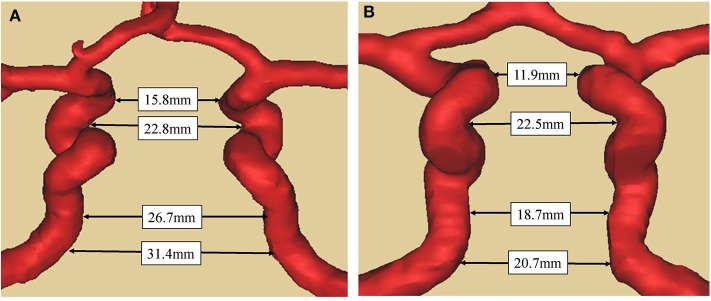
Comparison of bilateral ICAs distance between acromegaly and non-functional pituitary adenoma patient **(A)**. Case 1, a 59-year-old man with acromegaly. The tumor diameter is 1.8cm with knosp gradeII. The distance between the bilateral ICAs at a-d level (as in Figure 1) were respectively 15.8, 22.8, 26.7, and 31.4 mm. **(B)**. Case 2, a 61-year-old male with no-functional pituitary adenoma. The tumor diameter is 2.0cm with knosp gradeII. The distance between the bilateral ICAs at a-d level (as in Figure 1) were respectively 11.9, 22.5, 18.7, and 20.7 mm. The two patients have the same gender and similar age, and similar diameter of pituitary adenoma and same knosp grade.

Compared with patients in the control group, the maximum distance between bilateral siphon carotid ectasias (25.5 ± 4.1 vs. 23.4 ± 3.5 mm, *P* = 0.001) and distance between bilateral lacerum segments (26.2 ± 3.2 vs. 24.1 ± 4.3 mm, *P* < 0.001) were significantly higher for patients with acromegaly. On the other hand, the distance between bilateral ophthalmic segments—(16.0 ± 5.2 vs. 16.2 ± 3.4, *P* = 0.730) and the distance between bilateral cavernous segments (21.9 ± 3.2 vs. 22.5 ± 2.7, *P* = 0.205) were not significantly different between the two groups (Table 1). A typical illustration was showed in Figure 2.

### Determination of Related Factors of Change in ICAs Distance for Acromegalic Patients

The acromegaly group was divided into two subgroups by indicators with significant difference (distance between bilateral siphon carotid ectasias segments and distance between bilateral lacerum segments). After adding the two numbers together, patients were equally divided into large size group (*n* = 43) and small size group (*n* = 43). Six variables, including the age, volume of pituitary adenoma, diameter of pituitary adenoma, duration of the disease, atypical pituitary adenoma, and refractory pituitary adenoma had potential univariate associations (*p* < 0.15) with the large ICAs distance (Table 2) and were put into logistic regression model analysis. This analysis revealed that the refractory pituitary adenomas [odds ratio [OR] = 9.8, 95% confidence interval [CI] = 1.1–88.7, *P* = 0.043] and longer disease duration [OR = 1.01, 95%CI = 1.01–1.02, *P* = 0.005] were both the significant factors associated with the larger bilateral ICAs distance, while age, volume of pituitary adenoma, diameter of pituitary adenoma and did not show statistically significant difference (Table 3).

**Table 2:** Comparison of demographic and clinical data in acromegalic patients according to bilateral ICAs distance.

**Characteristics**	**Patients with larger bilateral ICA distance (*n* = 43)**	**Patients with smaller bilateral ICA distance (*n* = 43)**	***P*-value**
**DEMOGRAPHICS**			
Age, year	41.8 ± 10.8	38.4 ± 10.2	0.140
Gender, female	22 (51.2%)	18 (41.9%)	0.387
Height, cm	166.2 ± 5.6	168.3 ± 6.8	0.162
BMI, kg/m^2^	24.5 ± 4.6	25.1 ± 3.6	0.506
**MRI FOUNDING**			
Pituitary adenoma volume, cm^3^	3.7 (0.9, 6.8)	1.6 (0.6, 5.2)	0.107
Pituitary adenoma diameter, cm	1.9 ± 0.8	1.7 ± 0.7	0.146
Knosp grade	2 (1–3)	1 (1–3)	0.153
Grade 0	4	10	
Grade II	10	13	
Grade III	15	7	
Grade VI	11	9	
Grade V	3	4	
**LABORATORY**			
GH, μg/L	45.1 ± 36.0	49.9 ± 42.5	0.585
IGF-1, μg/L	511.2 ± 211.9	541.2 ± 198.2	0.520
**MEDICAL HISTORY**			
Hypertension	14 (32.6%)	9 (20.9%)	0.223
Diabetes mellitus	12 (27.9%)	7 (16.3%)	0.194
Hyperlipidemia	8 (18.6%)	6 (14.0%)	0.559
Somking history	8 (18.6%)	10 (23.3%)	0.596
Duration of disease, months	108 (72, 156)	72 (36, 96)	<0.001
Atypical pituitary adenoma	11 (25.6%)	3 (7.0%)	0.019
Refractory pituitary adenomas	8 (18.6%)	1 (2.3%)	0.030

*The acromegaly group was divided into two subgroups by indicators with significant difference (distance between bilateral siphon carotid ectasias segments and distance between bilateral lacerum segments). After adding the two numbers together, patients were equally distributed into two subgroups with larger numbers and smaller ones. Values are n (%), mean (SD), median (25%−75%)*.

**Table 3:** Logistic regression model analysis of larger bilateral ICA distance in acromegaly with possible factors^*^.

**Predictors**	**Univariate analysis**				**Multivariate analysis**^*******#*******^	
	**Patients with larger bilateral**	**Patients with smaller bilateral**	**OR (95% CI)**	***P*-value**	**OR (95% CI)**	***P*-value**
	**ICA distance (*n* = 43)**	**ICA distance (*n* = 43)**				
Age, yr	41.8 ± 10.8	38.4 ± 10.2	1.03 (0.99–1.08)	0.142	-	-
Pituitary adenoma volume, cm^3^	3.7 (0.9, 6.8)	1.6 (0.6, 5.2)	1.10 (0.98–1.24)	0.112	-	-
Pituitary adenoma diameter, cm	1.9 ± 0.8	1.7 ± 0.7	1.55 (0.86–2.79)	0.146	-	-
Duration of disease, months	108 (72, 156)	72 (36, 96)	1.01 (1.01–1.02)	0.002	1.01 (1.01–1.02)	0.005
Atypical pituitary adenoma	11 (25.6%)	3 (7.0%)	4.6 (1.2–18.0)	0.028	-	-
Refractory pituitary adenomas	8 (18.6%)	1 (2.3%)	9.6 (1.1–81.0)	0.037	9.8 (1.1–88.7)	0.043

*Values are n (%), mean (SD), median (25%−75%), OR = odds ratio*.

* *Among all the items belong to demographics, MRI finding, laboratory, medical history and duration of disease in Table 2, factors which had univariate associations P < 0.15 were included for further analysis*.

# *Multivariate analysis: factors with P < 0.05 in univariate analysis were included in multivariate analysis. Backward stepwise regression methods were used to produce the final model*.

## Discussion

Patients with acromegaly often undergo several treatments, which include transsphenoid or transcranial surgery, radiotherapy and several medical approaches. Transsphenoidal surgery is a first-line treatment for GH-secreting pituitary adenoma. Rupture of intracranial carotid artery during the surgery and formation of pseudoaneurysm after surgery were the common operative complications. Usually, these complications were caused by injury of the intracranial carotid artery during the surgery. Comparing to non-functional pituitary adenoma, functional pituitary adenoma has a higher risk of bleeding during the surgery (7). Furthermore, of pituitary adenomas, GH secreting tumors are considered at higher risk for ICA tear during surgery due to the higher occurrence of macroadenoma invading the neighboring cavernous sinus (10, 11). The impact of changed intercarotid distance on transsphenoidal surgery has been proven in several studies (12, 13). Banu et al. stated that the difference of intercarotid distance could affect the transsphenoidal angle and have further impact on the extent of tumor dissection (12). In their report, the volume of the sphenoid sinus and nare-sellar distance were proved to be correlated with intercarotid distance. That means change of intercarotid distance could affect neurosurgeon on the overview of basic cranial and surgical approaches. In addition, change of intercarotid distance has been demonstrated to be a predictive factor for surgical outcome and postoperative complications (13). Therefore, distances of the bilateral ICAs could not be ignored when planning for surgical strategy. For functional pituitary adenoma, those changes and characteristics may affect tumor resection and surgical safety to a great extent (14).

Because of excessive GH for a long time, morphology of ICAs in acromegalic patients has been changed. In addition, changes of vascular structure and function such as aortosclerosis, endothelial dysfunction, reconstruction of vessel walls and increased tortuosity of vessels exist in some patients (15, 16). According to comparison to patients with non-functional pituitary adenomas, our study aimed at determining changes of the distance between bilateral ICAs in acromegalic patients. We found that for the acromegalic patient group, the maximum distance between bilateral siphon carotid ectasias and distance between bilateral lacerum segments were significantly higher than those of the control group, respectively; and the distance between bilateral cavernous segments tended to be smaller for acromegalic patients, but the difference was not statistically significant. However, studies investigating the changes of ICAs distance in acromegalic patients have provided ambiguous results (5–7). In a study, Manara et al. reported different results comparing to ours. Based on image data of 177 acromegalic patients, they found a reduced intercarotid distance in C3 segment and increased intercarotid distance in C4 segment. Besides, Manara et al. selected subjects with headache or transient neurological deficits as their control group while we chose patients with non-functional pituitary adenomas. Mass effect of non-functional pituitary adenomas could affect the distance between bilateral ICAs because of compression. However, in contrast to acromegalic patients, there is no excess of GH secretion to stimulate the growth of various tissues that could impair the structures of ICAs. Thus, indicate that the GH exposure might impact the morphology of ICAs.

In the comparison between larger and smaller bilateral ICAs distance groups, the level of GH and IGF-1 were proved not to be the significant factor in the univariate analysis, whereas the duration of disease show significant different between these two groups in both univariate and multiple analysis. This means that the longer exposure duration of GH might be one of the key factors in changing the ICA morphology in patient with acromegaly. Meanwhile, in the univariate analysis, our study also revealed that atypical pituitary adenoma, refractory pituitary adenoma were significant factors associated with the occurrence of larger intercarotid artery distance, nevertheless, only refractory pituitary adenoma and duration of disease show statistically significant difference in the multivariate model. Therefore, refractory pituitary adenoma and longer disease duration with excessive GH might be the determining factors for the distance change of bilateral ICAs in acromegalic patients.

With the increasing application and importance of transsphenoidal surgery, anatomical structure of ICAs has drawn more attention in the past decades. Nowadays, transsphenoidal surgery has become a routine method to manage sellar tumors. The space-occupying lesion of hypophysial fossa, middle skull base and anterior skull base could affect anatomical structure of ICAs. Be aware of the morphology and anatomical variation of ICAs is the key to ensure surgical safety. Our study reported changes in the structure of ICAs, which was valuable to achieve greater security during the transsphenoidal surgery in acromegalic patients. For a long period of time in the past, bilateral ICAs and optic nerve have been considered as exclusion zone in the transsphenoidal surgery. With the obvious improvement of surgical techniques, surgeons could extend the approach to expose cavernous sinus, pterygopalatine fossa and other intracranial structure. ICAs was exposed to the operative field of vision and considered as an anatomical landmark to avoid injuries to other structure. Therefore, it is particularly important to determine the location and anatomical structure of ICAs before surgery.

Compared to patients with non-functional pituitary adenomas, acromegalic patients had significantly larger distances between bilateral siphon carotid ectasias, and between bilateral lacerum segments. These differences may be caused by local factors in combination with systematic factors. Mass effect is the main local factor for macroadenoma which is usually found in pituitary adenomas (17). The invasion of cavernous sinus and compression of ICAs in GH-secreting pituitary adenoma could expand the distance between bilateral cavernous segments of ICAs. However, some studies reported that invasion of cavernous sinus did not affect ICA morphology and found increased ICA tortuosity in microadenoma (5). Therefore, it is highly possible that ICA ectasia and tortuosity are not determined by vascular infiltration, mechanical effects or increased blood flow demand. In our study, there was no difference in diameter and Knosp grade of pituitary adenomas between experimental and control groups. However, the maximum distance between bilateral siphon carotid ectasias and distance between bilateral lacerum segments were significantly different between the two groups. The results further explained that local factors did not make a huge impact on the distances between bilateral ICAs in acromegalic patients.

Excessive growth hormone secretion and its related complications may be the systematic factors for the change in distance between bilateral ICAs in acromegalic patients. Growth hormone has shown a mitogenic effect on vascular endothelial cells, fibroblasts, macrophages and smooth muscle cells. It also plays a key role on the process of cell growth and metabolism (18, 19), which may affect the distance between bilateral ICAs. Moreover, collagen I to collagen III ratio could be altered by excessive GH, which has been suggested to be related to GH-dependent vascular alterations and aneurysm formation (20). That may be the reason for the changes of distances between bilateral ICAs in our study.

There were some limitations in our study. The current study is a single center retrospective study and has the known inherent limitations. We only compared bilateral ICAs distance according to CTA or MRA. The outcome and follow-up of subjects have not been linked to the change of distance. Further analysis should be conducted to confirm whether the change in distance is related to surgical outcome and postoperative complications. Further perspective study with large sample should be conducted to confirm whether the change in distance is related to surgical outcome and postoperative complications and investigate the clinical valuation of ICA distance as a tool in decision in acromegaly treatment.

## Conclusion

Our study showed significant change of the distance between the bilateral ICAs in patients with acromegaly. Compared to patients with non-functional pituitary adenomas, acromegalic patients had significantly larger distance between bilateral siphon carotid ectasias and distance between bilateral lacerum segments. The increased bilateral ICAs distance is associated with disease duration but not with the level of GH, IGF-1 or adenoma size. Refractory pituitary adenoma and longer duration of physiological stimulation by the obviously increased GH and IGF-1 might lead to the larger bilateral ICAs distance. Further large-scale studies are still needed to confirm this finding.

## Data Availability Statement

All datasets generated for this study are included in the article/supplementary material.

## Ethics Statement

The studies involving human participants were reviewed and approved by Ethics Committee of the First Affiliated Hospital of Fujian Medical University. The patients/participants provided their written informed consent to participate in this study. Written informed consent was obtained from the individual(s) for the publication of any potentially identifiable images or data included in this article.

## Author Contributions

ZL, XY, and CD revised the manuscript for important intellectual content. ZL, CJ, and CD take final responsibility for this article. All authors provided contributions to the study conception and design, acquisition of data or analysis and interpretation of data, drafting of the article, or revising it critically for important intellectual content, final approval of the version to be published, and analyzed and interpreted the data. All authors contributed to the article and approved the submitted version.

## Conflict of Interest

The authors declare that the research was conducted in the absence of any commercial or financial relationships that could be construed as a potential conflict of interest.
